# The acute-to-chronic glycemic ratio correlates with the severity of illness at admission in patients with diabetes experiencing acute ischemic stroke

**DOI:** 10.3389/fneur.2022.938612

**Published:** 2022-11-07

**Authors:** Chao Liu, Xu-ping Zhu, Xiao-wei Zhu, Yan-min Jiang, Guang-jun Xi, Lan Xu

**Affiliations:** ^1^Department of Endocrinology, The Affiliated Wuxi People's Hospital of Nanjing Medical University, Wuxi, China; ^2^Department of Geriatrics, Huadong Sanatorium, Wuxi, China; ^3^Department of Neurology, The Affiliated Wuxi People's Hospital of Nanjing Medical University, Wuxi, China

**Keywords:** acute-to-chronic glycemic ratio, acute ischemic stroke, severity of illness, acute hyperglycemia, diabetes

## Abstract

Acute hyperglycemia is a powerful indicator of the severity of acute ischemic stroke (AIS); however, the relationship between these two factors is not very clear in patients with diabetes. We aimed to retrospectively evaluate data from 335 consecutive patients who experienced AIS from November 2015 to November 2016 to investigate whether a comprehensive assessment of blood glucose levels is a more valuable indicator of the severity of AIS or the presence of acute hyperglycemia in patients with diabetes. We collected demographic data, clinical manifestation information, clinical scores, and laboratory data [including fasting blood glucose and glycated hemoglobin (HbA1c) levels]. We estimated prehospital mean blood glucose concentrations using the following formula [1.59 ^*^ HbA1c (%) – 2.59] to calculate the “Acute-to-Chronic Glycemic Ratio” (AC ratio). The AC ratio differed significantly among patients grouped according to the National Institutes of Health Stroke Scale/Score (NIHSS) at admission (admission NIHSS) (*p* = 0.006). Univariate regression analysis revealed a correlation between the AC ratio and admission NIHSS [standardized β-coefficient (Std. B) = 0.164, *p* = 0.004]. The adjusted linear regression analysis revealed a correlation between both HbA1c (Std. B = 0.368, *p* = 0.038) and the AC ratio (Std. B = 0.262, *p* = 0.022) and admission NIHSS. The AC ratio (Std. B = 0.161, *p* = 0.012) was related to admission NIHSS in the stepwise variable selection. For an admission NIHHS > 4, the AC ratio (Std. B = 0.186, *p* = 0.047) was related to admission NIHSS in the stepwise variable selection. The AC ratio (Std. B = 1.163, *p* = 0.006 and Std. B = 0.565, *p* = 0.021, respectively) were related to admission NIHSS in both large-artery atherosclerosis (LAA) and small-vessel occlusion (SVO) subgroups. Thus, the AC ratio is related to admission NIHSS in patients with diabetes who experienced AIS and may be a better indicator of severity than acute blood glucose levels.

## Introduction

Elevated levels of plasma glucose (acute hyperglycemia) are common in patients experiencing acute ischemic stroke (AIS), and acute hyperglycemia is an independent determinant of adverse outcomes (1–5) in patients with or without diabetes. Acute hyperglycemia promotes a state of thrombosis, drives inflammatory responses and oxidative stress, and results in cerebral dysfunction (3, 6–8); collectively, these changes can lead to greater infarct size and more severe neurological damage (3). For patients with diabetes, there is a weak correlation between mild blood sugar elevation and prognosis (5), and only when the blood glucose concentration is highly elevated, there exists a correlation between blood glucose levels and AIS (9). These findings suggest that acute hyperglycemia may not be directly related to the severity of AIS in patients with diabetes, as the pre-existing dysregulation of blood glucose levels may be a confounder. For example, the concentrations of hemoglobin A1c (HbA1c) were found a determinant of adverse consequences related to AIS (10, 11). In such patients, the presence of acute hyperglycemia might be a more potent indicator of illness severity if the effects driven by the previous dysregulation of blood glucose levels could be controlled.

Historically, fasting glucose levels have often been used to infer the degree of elevation of blood glucose concentrations. However, there have been some studies (12–16) that instead used the “Acute-to-Chronic Glycemic Ratio (AC ratio)”, as this factor correlated well with illness severity in patients with acute myocardial infarction and infection, and the use of this processed ratio appeared to be a better indicator of the “real” elevated blood glucose response. Thus, the purpose of our study was to investigate the possible relationship between the AC ratio and the severity of AIS in patients with diabetes.

## Materials and methods

### Patients

We included all consecutive patients with type 2 diabetes who developed and were treated for AIS from November 2015 to November 2016 in the Department of Neurology of Wuxi People's Hospital affiliated with Nanjing Medical University (*n* = 335). The inclusion criteria of patients with AIS were as follows: (1) occurrence of ischemic stroke symptoms within the 72 h prior to admission; and (2) a diagnosis of AIS based on magnetic resonance imaging (MRI) or computed tomography (CT). Patients meeting the following criteria were excluded: (1) patients experiencing definitive non-ischemic neurological deficits, such as hemorrhagic stroke; (2) patients experiencing transient ischemic attack; and (3) patients whose symptoms were attributed to causes, such as vasculitis, moyamoya disease, or tumor-related stroke. Stroke etiology was classified according to the Trial of Org 10172 in Acute Stroke Treatment (TOAST) criteria after complete diagnostic profiling (17).

### Research design

The included patients were admitted to the ward within 24 h, and clinical biochemical examinations were subsequently conducted. Admission glucose is defined as the first blood glucose test result before admission to the ward. On the first day of the stay in the ward, fasting glucose, glycated hemoglobin, urea nitrogen, C-reactive protein (CRP), albumin, serum creatinine, and cholesterol levels, among other factors, were recorded. The age, sex, and history of diseases were also recorded for each patient. Demographic, clinical, and biochemical data were measured within 24 h after admission.

Type 2 diabetes was confirmed in each case when the hospital medical records indicated a disease diagnosis or if the patient had undergone treatment with antidiabetic agents (including oral medications or insulin). According to published guidelines, an attending physician initiated standard medical treatment for each patient. During admission, no antidiabetic medication was administered to all patients with diabetes, and the target range of blood glucose levels was 8–10 mmol/L. The prehospital average glucose level was estimated by HbA1c according to the following formula as previously described (18):

Estimated prehospital average glucose level (mmol) = 1.59^*^HbA1c (%) - 2.59.

Subsequently, the AC ratio was calculated as follows:

AC ratio = fasting blood glucose level / estimated prehospital average glucose level.

In each patient, the National Institutes of Health Stroke Scale/Score NIHSS (19) was calculated at admission and before discharge.

### Ethics and consent

All research documents and procedures were approved by the Ethics Committee of Wuxi People's Hospital affiliated with Nanjing Medical University. All patients or their designated relatives provided written informed consent before participation in the study. No patient who participated in the study experienced a delay in treatment.

### Statistical analysis

One-way analysis of variance (ANOVA) was used to compare the differences between groups for continuous variables with a normal distribution. The nonparametric Kruskal-Wallis rank-sum test was used to compare the differences between groups for continuous variables with the nonnormal distribution. For categorical variables, a chi-square test was used for group comparisons. Subsequently, the relationship between the AC ratio and admission NIHSS was evaluated by regression analysis. Single-factor linear regression analysis, multi-factor linear regression analysis, and stepwise regression analysis were conducted to study the relationship between independent variables and admission NIHSS. All tests were two-tailed, and *p*<0.05 indicated statistical significance. All analyses were performed using Statistical Package for the Social Sciences (SPSS) Statistics version 19 software (IBM Corp., Armonk, NY, USA).

## Results

A total of 355 consecutive patients with AIS (mean age: 68.2 ± 11.1 years) were included in the study; of whom, 207 were male. All included patients had been diagnosed with diabetes. Overall, the mean fasting blood glucose concentration was 8.20 ± 2.99 mmol/L, the mean admission glucose concentration was 10.77 ± 5.11 mmol/L, the mean HbA1c level was 8.00 ± 1.95%, and the mean AC ratio was 0.84 ± 0.25.

Table 1 shows the baseline characteristics of the different patient groups classified according to admission NIHSS. The different groups comprised patients of similar ages and similar percentages of patients with hypertension, coronary heart disease, stroke, and smoking history. There were also no differences in fasting blood glucose, admission glucose, and HbA1c levels (*p* = 0.786, *p* = 0.315, and *p* = 0.143, respectively). Patients with higher admission NIHSS values had higher AC ratios than patients with lower admission NIHSS values (*p* = 0.006).

**Table 1:** Baseline characteristics of different groups separated according to admission NIHSS.

	**Admission NIHSS**						***p***
	**0–2**	**3–4**	**5–6**	**7–8**	**9–10**	**>10**	
N	83	79	76	37	34	26	
Age (years)	68.69 ± 11.08	66.06 ± 12.04	68.09 ± 11	69.46 ± 10.67	70.5 ± 10.05	68.46 ± 10.37	0.413
Male, sex	55 (66.3%)	54 (68.4%)	48 (63.2%)	23 (62.2%)	18 (52.9%)	9 (34.6%)	0.043
Hypertension	71 (85.5%)	71 (89.9%)	67 (88.2%)	33 (89.2%)	30 (88.2%)	23 (88.5%)	0.977
Coronary heart disease	7 (8.4%)	9 (11.4%)	5 (6.6%)	2 (5.4%)	2 (5.9%)	1 (3.8%)	0.751
History of Stroke	17 (20.5%)	24 (30.4%)	19 (25%)	12 (32.4%)	8 (23.5%)	12 (46.2%)	0.163
Atrial fibrillation	5 (6%)	2 (2.5%)	2 (2.6%)	1 (2.7%)	0 (0%)	2 (7.7%)	0.480
Smoking	14 (16.9%)	20 (25.3%)	13 (17.1%)	6 (16.2%)	4 (11.8%)	1 (3.8%)	0.179
NIHSS at admission	1 (1, 2)	3 (3, 4)	5 (5, 6)	8 (7, 8)	9.5 (9, 10)	14 (11, 16)	<0.001
NIHSS on discharge	1 (1, 2)	3 (2, 3)	4 (3, 5)	6 (5.5, 7)	8 (7, 9)	12 (10, 15)	<0.001
LAA	28 (33.7%)	28 (35.4%)	33 (43.4%)	15 (40.5%)	19 (55.9%)	5 (19.2%)	0.021
CE	5 (6.0%)	2 (2.5%)	2 (2.6%)	1 (2.7%)	0 (0%)	2 (7.7%)	
SVO	33 (39.8%)	37 (46.8%)	30 (39.5%)	17 (45.9%)	11 (32.4%)	7 (26.9%)	
SOE	0 (0%)	0 (0%)	0 (0%)	0 (0%)	0 (0%)	0 (0%)	
SUE	17 (20.5%)	12 (15.2%)	11 (14.5%)	4 (10.8%)	4 (11.8%)	12 (46.2%)	
Fasting glucose (mmol/L)	7.98 ± 2.84	8.28 ± 3.44	8 ± 2.72	8.47 ± 3	8.28 ± 3.24	8.9 ± 2.51	0.786
Admission glucose (mmol/L)	11.58 ± 4.22	12.53 ± 4.1	13.36 ± 5.02	12.31 ± 5.48	14.93 ± 7.53	12.49 ± 4.88	0.143
HbA1c (%)	7.92 ± 1.97	7.99 ± 1.83	8.08 ± 1.84	7.87 ± 1.45	8.67 ± 2.75	7.43 ± 1.99	0.315
Hemoglobin (g/L)	132.86 ± 16.84	130.51 ± 17.08	134.47 ± 14.79	130.97 ± 18.53	128.52 ± 16.54	127.8 ± 23.02	0.411
AC ratio	0.816 (0.685, 0.928)	0.812 (0.662, 0.93)	0.794 (0.688, 0.922)	0.87 (0.734, 1.017)	0.801 (0.585, 0.957)	0.983 (0.89, 1.111)	0.006
CRP (mg/L)	6 (4.5, 9)	6 (4, 14.5)	6 (4, 12)	5 (4, 10.3)	11.5 (5, 24.3)	19 (9.5, 29.5)	0.001
BUN (mmol/L)	5.4 (4.6, 6.75)	5.6 (4.6, 6.85)	5.6 (4.95, 6.75)	6 (4.8, 6.75)	6.1 (4.75, 7.95)	6.2 (4.3, 11.4)	0.672
Cr (μmol/L)	64.35 (55, 85.18)	66.3 (59.05, 81.8)	72.1 (59.5, 85.9)	69.7 (62.35, 80.1)	69 (57.4, 91.15)	68.2 (56.45, 96.6)	0.943
Bilirubin (μmol/L)	16.37 ± 6.4	15.94 ± 6.72	16.19 ± 4.92	13.1 ± 4.39	17.07 ± 9.11	15.89 ± 6.46	0.483
Albumin (g/L)	42.09 ± 3.47	43.73 ± 4	42.39 ± 4.91	40.93 ± 5.62	42.04 ± 4.62	40.89 ± 3.87	0.323
TC (μmol/L)	4.21 ± 0.94	4.32 ± 1.24	4.37 ± 1.06	4.45 ± 1.05	4.41 ± 1.28	5 ± 1.77	0.138
LDL-C (μmol/L)	2.53 ± 0.8	2.6 ± 1	2.69 ± 0.96	2.83 ± 0.92	2.58 ± 0.89	3 ± 1.8	0.375
Fibrinogen (g/L)	3.03 ± 0.86	3.04 ± 0.73	3 ± 0.89	3.18 ± 1.05	2.87 ± 0.89	3.28 ± 1.23	0.632
Homocysteine (μmol/L)	16.36 ± 6.53	14.96 ± 7.35	15.73 ± 6.54	15.64 ± 6.04	17.74 ± 10.48	15.66 ± 4.18	0.746

Data are presented as mean ± standard deviation or median (IQR) or n (%).

NIHSS, National Institutes of Health Stroke Scale; HbA1c, hemoglobin A1c; CRP, C-reactive protein; BUN, blood urea nitrogen; Cr, plasma creatinine; TC, total cholesterol; LDL-C, low-density lipoprotein cholesterol; LAA, large-artery atherosclerosis; CE, cardioembolism; SVO, small-vessel occlusion; SOE, stroke of other determined etiology; SUE, stroke of undetermined etiology.

Table 2 shows the results of the regression analysis based on admission NIHSS. The univariate analysis revealed that the AC ratio [standardized β-coefficient (Std. B) = 0.164, *p* = 0.004] was related to the admission NIHSS, but not to fasting blood glucose or HbA1c levels. HbA1c (Std. B = 0.368, *p* = 0.038) and the AC ratio (Std. B = 0.262, *p* = 0.022) were related to admission NIHSS after adjusting for major clinical confounding factors (age, sex, previous stroke history, CRP, and admission glucose levels). The linear regression analysis model showed that sex (Std. B = −0179, *p* = 0.005), history of stroke (Std. B = 0.143, *p* = 0.027), and the AC ratio (Std. B = 0.161, *p* = 0.012) were related to admission NIHSS in the stepwise variable selection process.

**Table 2:** Regression analysis of admission NIHSS.

	**Unadjusted**			**Adjusted**			**Stepwise adjusted**		
	**B**	**Std. B**	***p***	**B**	**Std. B**	***p***	**B**	**Std. B**	***P***
Age (years)	0.019	0.055	0.312	0.01	0.028	0.673			
Sex (compare to female)	−1.257	−0.164	0.003	−1.417	−0.176	0.007	−1.439	−0.179	0.005
History of Stroke	1.154	0.138	0.011	1.316	0.155	0.019	1.213	0.143	0.027
CRP(mg/L)	0.03	0.186	0.003	0.02	0.115	0.073			
Fasting glucose (mmol/L)	0.098	0.079	0.159	−0.414	−0.292	0.106			
Admission glucose (mmol/L)	0.069	0.094	0.162	−0.028	−0.031	0.718			
HbA1c (%)	−0.003	−0.001	0.979	0.751	0.368	0.038			
AC ratio	2.403	0.164	0.004	4.08	0.262	0.022	2.418	0.161	0.012

NIHSS, National Institutes of Health Stroke Scale; HbA1c, hemoglobin A1c; CRP, C-reactive protein; B, regression coefficient; Std. B, standardized regression coefficient; AC ratio, Acute-to-Chronic Glycemic Ratio.

Table 3 shows the results of the regression analysis based on layered admission NIHSS values. At the admission NIHHS of ≤ 4 level, no variables exhibited a statistically significant relationship (including HbA1c and AC ratio) in the regression analysis. At the admission NIHHS of >4 level, a history of stroke was related to admission NIHSS in the adjusted regression analysis. The linear regression analysis model revealed that a history of stroke (Std. B = 0.216, *p* = 0.021) and the AC ratio (Std. B = 0.186, *p* = 0.047) were related to admission NIHSS in the stepwise variable selection process.

**Table 3:** Regression analysis of admission NIHSS at different levels.

	**NIHSS** ≤ **4**			**NIHSS**>**4**					
	**Adjusted**			**Adjusted**			**Stepwise Adjusted**		
	***B***	**Std. B**	***p***	***B***	**Std. *B***	***p***	***B***	**Std. *B***	***p***
Age(years)	−0.007	−0.069	0.483	−0.027	−0.08	0.415			
Sex(compare to female)	−0.013	−0.005	0.956	−0.983	−0.141	0.143			
History of Stroke	0.365	0.133	0.164	1.645	0.223	0.027	1.599	0.216	0.021
CRP(mg/L)	0.005	0.093	0.333	0.017	0.111	0.253			
Fasting glucose (mmol/L)	0.239	0.627	0.193	−0.225	−0.177	0.4			
Admission glucose (mmol/L)	0.027	0.073	0.53	−0.1	−0.145	0.29			
HbA1c (%)	−0.293	−0.465	0.263	0.552	0.328	0.17			
AC ratio	−2.279	−0.415	0.233	4.805	0.409	0.082	2.184	0.186	0.047

NIHSS, National Institutes of Health Stroke Scale; HbA1c, hemoglobin A1c; CRP, C-reactive protein; B, regression coefficient; Std. B, standardized regression coefficient; AC ratio, Acute-to-Chronic Glycemic Ratio.

Table 4 shows the results of the regression analysis in different etiology subgroups. In the LAA subgroup, HbA1c (Std. B = 1.092, *p* = 0.006) and the AC ratio (Std. B = 1.163, *p* = 0.006) were related to admission NIHSS after adjusting for major clinical confounding factors (age, sex, previous stroke history, CRP, and admission glucose levels). In the SVO subgroup, the AC ratio (Std. B = 0.565, *p* = 0.021) was related to admission NIHSS after adjusting for major clinical confounding factors. In the SUE subgroup, no indicators show the correlation.

**Table 4:** Regression analysis of admission NIHSS in different etiology subgroups.

	**LAA**			**SVO**			**SUE**		
	**B**	**Std. B**	***p***	**B**	**Std. B**	***p***	**B**	**Std. B**	***p***
Age (years)	0.025	0.077	0.455	−0.047	−0.126	0.279	0.089	0.278	0.096
Sex (compare to female)	−0.527	−0.075	0.48	−1.85	−0.253	0.02	−1.763	−0.169	0.287
History of Stroke	0.288	0.037	0.739	1.933	0.262	0.014	0.565	0.057	0.719
CRP(mg/L)	0.021	0.134	0.198	0.012	0.07	0.494	0.011	0.053	0.753
Fasting glucose (mmol/L)	−1.46	−1.186	0.012	−0.635	−0.485	0.046	1.538	0.964	0.145
Admission glucose (mmol/L)	0.06	0.064	0.631	−0.029	−0.042	0.784	0.174	0.129	0.495
HbA1c (%)	2.102	1.092	0.006	0.567	0.326	0.209	−1.162	−0.48	0.381
AC ratio	17.319	1.163	0.006	7.055	0.565	0.021	−8.641	−0.526	0.427

NIHSS, National Institutes of Health Stroke Scale; HbA1c, hemoglobin A1c; CRP, C-reactive protein; B, regression coefficient; Std. B, standardized regression coefficient; LAA, large-artery atherosclerosis; SVO, small-vessel occlusion; SUE, stroke of undetermined etiology; AC ratio, Acute-to-Chronic Glycemic Ratio.

## Discussion

The main finding of this study is that the AC ratio can be used to assess the AIS status of patients with diabetes and may be a better indicator than fasting blood glucose and HbA1c levels when evaluating illness severity in patients with AIS.

Acute hyperglycemia is frequently observed in the early phase of AIS (3, 20), irrespective of the concomitant presence of diabetes, and it has been consistently shown to be associated with a poor outcome and larger infarct size (3, 6, 8). Some studies (21, 22) found that hyperglycemia could aggravate cerebral edema and increase the risk of early neurological deterioration. Changes in the levels of stress hormones (23), such as cortisol (24) and catecholamines (25), worsening of the state of thrombosis (7, 26), increased inflammatory responses, and oxidative stress might be involved in the hyperglycemia phenomenon. A variety of inflammatory factors including interleukin-6 (IL-6), matrix metalloproteinase-9 (MMP-9), tumor necrosis factor α (TNF-α), and hypersensitive C-reactive protein (hs-CRP), increased among patients with AIS (27). These changes may activate thioredoxin interacting protein(TXNIP), NF-κB pathway, etc., and damage the neurons (28, 29).

The impact of acute hyperglycemia is more pronounced in patients without diabetes than in those with the disease (4, 5, 30). Clinical observations (1, 4, 9, 30) have shown that in patients with diabetes experiencing AIS, the blood glucose level was related to illness severity only when it was significantly higher than that of patients without diabetes. This difference suggested that the magnitude of the increase above chronic levels in acute hyperglycemia, rather than the absolute admission glycemic level *per se*, can be detrimental. Therefore, we hypothesized that the ratio of the acute and chronic blood glucose levels would be better than the absolute fasting blood glucose value measured at the time of hospital admission for identifying true stress-induced hyperglycemia. To estimate the average degree of chronic glycemia, we chose to measure the HbA1c level, which reflected the blood glucose situation over the previous 8–12 weeks, and we employed the formula proposed by Nathan et al. (18).

To the best of our knowledge, this is one of the first studies (14, 31–33) to explore the role of the AC ratio in patients with AIS. While the prognostic impact of glycemia has been widely evaluated in those with AIS, the clinical relevance of the AC glycemic ratio has not been fully investigated. A very recent study by Chen et al. (33) analyzed the association between the AC ratio (defined in their study as the stress hyperglycemia ratio) and clinical outcomes at 3 months in 160 patients undergoing mechanical thrombectomy to treat proximal acute occlusion in the anterior circulation, showing that an increased AC ratio was strongly correlated with a poor outcome. However, contrary to our study, they did not distinguish between patients with or without diabetes in their population, and only 18.1% of participants were diabetic. Another study by Yang et al. (31)investigated whether HbA1c-based adjusted glycemic variables were associated with unfavorable outcomes among patients admitted to the hospital for AIS. However, their ratio was directly related to the HbA1c level, which was used as a denominator in their formula. In our study, we used the formula proposed by Nathan et al. (18) to calculate the estimated prehospital average glucose level, which was then used to calculate the AC ratio.

We found that in patients with different admission NIHSS levels, there were no statistically significant differences in fasting glucose, admission glucose, or HbA1c levels, although the AC ratio was significantly different. The single-variable linear regression analysis led to similar conclusions, with only the AC ratio showing a correlation with admission NIHSS. After adjusting for certain confounding factors, the fasting glucose and HbA1c levels showed a statistical correlation with admission NIHSS. These findings are similar to those of previous research (1–5). Blood glucose and HbA1c levels, the AC ratio, and other potential independent variables were evaluated by linear regression using the stepwise method of analysis to identify those with a significant impact on admission NIHSS, with the goal of constructing the optimal regression equation. The results showed that, relative to the glycemic index, the AC ratio might be a better indicator of admission NIHSS-related outcomes.

We evaluated whether the AC ratio had a better performance as an indicator at certain NIHSS levels. In the case of NIHSS of ≤ 4, no statistical significance was observed for any of the potential independent variables. For an admission NIHSS of >4, the results of the linear regression analysis showed no statistically significant relationship for the HbA1c level or the AC ratio; however, after employing the gradual stepwise analysis method, the AC ratio did exhibit a statistically significant relationship. We speculate that in patients with relatively mild diseases (i.e., those with an NIHSS of ≤ 4), various factors could slightly affect their clinical condition. However, to evaluate the effects of inflammatory indicators, blood glucose and glycated hemoglobin levels, and other influencing factors, it may be necessary to increase the number of subjects included in the analysis to ensure sufficient statistical power to observe a significant effect. However, for patients with more severe illness (NIHSS of >4), a potential correlation with the AC ratio is more likely, as a trend toward significance was observed (*p* = 0.082). Finally, statistical significance was observed for the AC ratio in the stepwise regression analysis in which the ratio competed with other potential indicators.

For other blood glucose-related indicators, such as glycated hemoglobin, the full NIHSS regression analysis revealed statistical correlations. For example, the standardized regression coefficient was slightly higher for HbA1c than for the AC ratio (Std. B = 0.368 and 0.262, respectively), indicating that HbA1c is also a relatively good indicator. In the subsequent stepwise method of variable selection, however, HbA1c was not selected, which suggested that the AC ratio may be a better indicator than HbA1c.

We have observed that among patients with relatively mild illness, HbA1c and the AC ratio are not significantly related. Among patients with relatively serious illnesses, HbA1c was eliminated during the stepwise regression process. We speculate that there were several possibilities that could explain this result. The first possibility is that HbA1c is a long-term indicator of chronic conditions, whereas the AC ratio may better reflect the manifestation of acute changes related to certain diseases (34). The second is that HbA1c is also a necessary factor, and overall, a decrease in the number of patients with a relatively severe clinical condition may lead to differences in statistical significance. Third, in the multiple linear regression analysis, HbA1c (*p* = 0.17) was higher than that of the AC ratio (*p* = 0.082), a result that may be related to the greater competitiveness of the AC ratio in the subsequent stepwise regression analysis.

We also observed the AC ratio in different Toast subgroups. Our research eliminated AIS patients with a determined etiology, so there was no patient in the SOE group. For the CE group, because there were only 12 patients, we mainly focused on the three groups of LAA, SVO, and SUE. We found that in the LAA and SVO groups, the AC ratio can perform the correlation, but the SUE group does not show the correlation. This result prompted that there may be other potential factors for patients with AIS with undetermined etiology, which may need a larger sample volume to explore this phenomenon.

Our research has some limitations. The first limitation is that this is a cross-sectional study without a follow-up period, making it difficult to assess the relationship between the AC ratio and patient prognosis over time. Also, the number of patients included in the study was small, and the subgroups were smaller still after categorizing the patients according to admission NIHSS. Finally, despite taking into account the history of cerebral infarction, we could not completely rule out its effect on the current condition of the patient.

## Conclusion

Our study may have some clinical significance. The AC ratio can be used to identify “real” acute hyperglycemia, it can assess the condition of patients with diabetes who have experienced AIS, and it may be a better indicator of the severity of AIS than fasting blood glucose and glycated hemoglobin levels.

## Data availability statement

The original contributions presented in the study are included in the article/supplementary material, further inquiries can be directed to the corresponding authors.

## Ethics statement

The studies involving human participants were reviewed and approved by the Ethics Committee of Wuxi People's Hospital. Written informed consent for participation was not required for this study in accordance with the national legislation and the institutional requirements.

## Author contributions

LX and G-jX conceived, designed, and coordinated the experiments. CL and X-pZ carried out the research, collected and sorted out data, and drafted and wrote the manuscript. X-wZ and Y-mJ revised the manuscript. All authors have read and agreed to the published version of the manuscript.

## Funding

This work was supported by the Major Project of Wuxi Health Planning Commission, China (Z201807) and the General project of Wuxi Science and Technology Bureau, China (N20202006).

## Conflict of interest

The authors declare that the research was conducted in the absence of any commercial or financial relationships that could be construed as a potential conflict of interest.

## Publisher's note

All claims expressed in this article are solely those of the authors and do not necessarily represent those of their affiliated organizations, or those of the publisher, the editors and the reviewers. Any product that may be evaluated in this article, or claim that may be made by its manufacturer, is not guaranteed or endorsed by the publisher.
